# Inconsistency in legal liability in Article 9G of law number 1 of 2025 concerning state-owned enterprises

**DOI:** 10.3389/fsoc.2026.1791433

**Published:** 2026-06-23

**Authors:** Mirza Nasution

**Affiliations:** University of North Sumatra, Medan, Indonesia

**Keywords:** accountability, article 9G, inconsistency, law, state-owned enterprises

## Abstract

**Aim:**

The establishment of BUMN (State-Owned Enterprises) involved in the management of state-owned enterprises aims to contribute to national economic development and state revenue, provide high-quality goods and services to meet the needs of the community, and become a pioneer in business fields that have not been widely managed by the private sector. Problems related to losses in BUMN occur due to negligence and mismanagement by the Board of Directors. Eliminating criminal liability for directors who cause losses to BUMN can create opportunities for crimes and criminal acts that further cause losses to BUMN. The importance of this study is to analyze the limits of directors’ actions that cannot be punished criminally and this study aims to evaluate the institutional consequences of violations committed by directors in BUMN.

**Subject and methods:**

The enactment of Law Number 1 of 2025 concerning State-Owned Enterprises, which states that the Board of Directors is not a state administrator, has sparked debate over the legal accountability of the Board of Directors. The research method used focusses on the legal norms, rules, and principles found in legislation or other legal sources. This research approach uses a case study approach aimed at analysing the rules and their application, as well as the sanctions imposed.

**Results:**

The entire data will be analysed to find solutions to the existing problems. The research results explain that legal liability, whether criminal, civil, or administrative, can be applied as long as the Board of Directors is proven to have committed the act.

**Discussion:**

Harmonisation of laws related to the management of BUMN must be carried out, particularly concerning the status of BUMN as legal subjects equal to other legal subjects, namely individuals and private corporations, to ensure legal certainty in the application of the law regarding the legal accountability of the Board of Directors.

## 1 Introduction

The operation of State-Owned Enterprises is based on economic democracy, which includes: (a) collectivity, (b) fair efficiency, (c) sustainability, (d) environmental awareness, (e) maintaining balance, progress, and national economic unity; and (f) good corporate governance. Good corporate governance is manifested in the form of plans, processes, and results that can be accounted for by the managers of the State-Owned Enterprise. BUMN managers must also provide opportunities, support, protection, and partnerships in the development of micro, small, and medium enterprises and cooperatives as the main pillars of national economic development (Indonesia, 2008). The management elements within BUMN have been regulated as stated in Article 3, which explains, “The Minister, as the representative of the Central Government and the regulator, is tasked with establishing policies, regulating, fostering, coordinating, and supervising the implementation of BUMN management policies.” In order to ensure dividend contributions for investment, management, the Minister places their representatives in the Agency, the Investment Holding, and the operational Holding with the President’s approval. Article 3AA states that unless specifically regulated in this Law, the provisions of laws and regulations governing the management of state finances separated at BUMN the state treasury, non-tax state revenues, and limited liability companies shall not apply to the Body. Article I “State-Owned Enterprises, hereinafter referred to as BUMN, are business entities that meet at least one of the following conditions”:

a) all or most of its capital is owned by the Republic of Indonesia through direct participation; or
b) There are privileges that the Republic of Indonesia possesses.

Article 9G “Members of the Board of Directors, Board of Commissioners, and Supervisory Board of State-Owned Enterprises are not state officials.” Article 3Y “The Minister, organs, and employees of the Agency cannot be held legally accountable for losses if they can prove”:

a) The loss was not due to his fault or negligence;
b) Has managed with good faith and care in accordance with the purpose and objectives of the investment and governance;
c) Does not have any conflict of interest, either direct or indirect, regarding investment management actions; and
d) Not obtaining personal gain illegally.

Article 9F: (1) “Members of the Board of Directors cannot be held legally liable for losses if they can prove”:

a) The loss was not due to his fault or negligence;
b) Has managed with good faith and care for the interests and in accordance with the objectives of the BUMN;
c) Does not have any direct or indirect conflict of interest regarding management actions that result in losses; and
d) Has taken action to prevent the occurrence or continuation of such losses.

Article 9F: (2) “Members of the Board of Commissioners or the Supervisory Board of BUMN cannot be held liable for losses if they can”:

a) Has carried out supervision in good faith and with due care for the benefit of the state-owned enterprise and in accordance with the state-owned enterprise’s objectives;
b) Has no personal interest, either directly or indirectly, in the management actions of the Board of Directors that result in losses; and
c) Has provided advice to the Board of Directors to prevent the occurrence or continuation of such losses.

In its explanation, it mentions Article 9G: “It is not interpreted to mean that if someone who is not a state official becomes a manager of a state-owned enterprise, their status as a state official will be lost.” The status of non-state organiser toward the Board of Directors as stipulated by Law Number 1 of 2025 concerning State-Owned Enterprises may preclude the possibility of legal accountability for the actions of the directors that cause losses to the State-Owned Enterprise (Adebayo, 2025). Legal accountability, whether criminal, civil, or administrative, can be applied as long as the Board of Directors is proven to have committed the actions. Harmonisation of laws related to the management of BUMN must be carried out to ensure legal certainty in cases of violations committed by the Board of Directors. The inconsistencies in Article 9 Letter G of the State-Owned Enterprises Law can be explained as follows:

a) According to the Corruption Eradication Commission (KPK) and several legal observers, Article 9G contradicts the definition of state administrators stipulated in Law No. 28 of 1999 concerning Clean and Corruption-Free State Administration, Collusion-Free, and Nepotism (KKN). Under this law, directors, commissioners, and structural officials of BUMN are included in the category of state administrators, so certain legal obligations (such as reporting LHKPN or accepting gratuities) remain in effect;
b) The KPK Law (Law No. 19/2019) stipulates that the object of KPK action is state administrators who commit acts of corruption. By declaring BUMN managers not state administrators, this provision has the potential to narrow the scope of the KPK’s authority in handling corruption in BUMN, although the KPK itself emphasizes that the application of the State Administrators Law remains relevant and that the explanation of Article 9G implies that the status of state administrators does not simply disappear;
c) The explanation of Article 9G, which states that the status of state administrator is not interpreted as being lost for BUMN managers, actually demonstrates an internal inconsistency. On the one hand, the text of the article revokes that status, but on the other hand, the explanation of the article allows for its continued retention under other laws. This has the potential to create legal uncertainty and differing interpretations by law enforcement officials and the courts.

## 2 Literatur review

### 2.1 The main content of Article 9G and the paradigm shift in accountability

Substantially, Article 9G of the State-Owned Enterprises Law states that “members of the Board of Directors, the Board of Commissioners, and the Supervisory Board of State-Owned Enterprises are not state administrators.” This provision differs from the previous regime where state-owned enterprise management was explicitly considered state administrators in the context of public financial management. Regarding this change, some literature highlights a new paradigm in legal accountability and the status of BUMN losses: (a) The new law separates BUMN losses from state losses and protects the Board of Directors from liability if decisions are made in good faith according to the principle of the Business Judgement Rule (BJR), (b) This change introduces legal uncertainty because it deviates from the legal treatment of BUMN as public entities toward a corporate legal regime.

### 2.2 Between the state-owned enterprises law and other laws

That the provisions of Article 9G are contradictory to the rules in: (a) Law Number 28 of 1999 concerning the Clean and Corruption-Free Governance of the State, which traditionally includes BUMN managers in the definition of state officials, (b) the State Finance Law which states that BUMN assets remain part of state finances.

### 2.3 Implications for law enforcement: the KPK and BUMN corruption

That Article 9G can lead to multiple interpretations regarding the scope of law enforcement: (a) Article 9G is considered to change the scope of authority of the Corruption Eradication Commission (KPK) because the KPK has so far been authorised to investigate corruption involving state officials, law enforcement officers, and other related parties in accordance with the KPK Law, (b) This ambiguity raises concerns that corruption cases in state-owned enterprises can be processed outside the public corruption criminal act regime, for example, simply under general criminal provisions, (c) Conversely, another view (e.g., a statement from the KPK) asserts that the KPK remains authorised, because corruption criminal acts can also be classified as “any person” and not just state officials, and if there is an unlawful act that harms the state, the KPK can still take action.

The study in this research uses the theory of legal certainty to examine its application to law enforcement in accordance with applicable laws and regulations. The idea of this principle of legal certainty was initially introduced by Gustav Radbruch in his book titled “Einführung in die Rechtswissenschaften.” Radbruch wrote that there are 3 (three) fundamental values in law, namely: (1) Justice (Gerechtigkeit); (2) Utility (Zweckmassigkeit); and (3) Legal Certainty (Rechtssicherheit). In his book “The Science of Law,” Satjipto Rahardjo illustrates these three basic values with the foundation of their validity. The ragas are as follows:

The principle of legal certainty, in essence, is understood as a state where the law is certain due to the concrete force of the law in question (Braithwaite, 2002). The existence of the principle of legal certainty is a form of protection for litigants (justice seekers) against arbitrary actions, meaning that a person will and can obtain what is expected under certain circumstances (Julyano and Sulistyawan, 2019).

Corruption cases involving state-owned enterprise (BUMN) directors lead to failures in BUMN management and cause losses to the state. Corruption results in financial losses, hinders economic growth, reduces investment, increases poverty, and exacerbates social inequality. On the other hand, BUMN directors can also be held accountable if they engage in actions that harm state finances, although there is debate about whether BUMN losses are equivalent to state financial losses.

The board of directors of state-owned enterprises can be held accountable for losses if proven guilty or negligent in performing their duties, despite provisions that protect directors who act in good faith in carrying out their responsibilities (Thompson and Alleyne, 2023).

## 3 Methodology

The research methodology used focuses on legal norms, rules, and principles contained in laws or other legal sources. This research approach uses a case study method, which aims to analyze the rules and their application, as well as the sanctions imposed. All data will be analyzed to find solutions to existing problems. The analytical methodology is doctrinal by examining laws and several court decisions supported by a hermeneutic approach to find the original meaning of Article 9G of Law Number 1 of 2025 concerning State-Owned Enterprises. Several sample cases used are several cases of BUMN directors who have been sentenced to criminal penalties for state losses incurred in BUMN and this case aims to observe the level of impact of state losses if the violations committed by BUMN directors cannot be classified as criminal acts. The data used will serve as material for in-depth analysis to reformulate appropriate norms to prevent state losses. The sample indicators used include: (1) companies that commit violations and have received sanctions, (2) directors or commissioners of companies who have been punished for violations that cause company losses.

## 4 Discussion

### 4.1 Status of BUMN directors not as state administrators

The board of directors of State-Owned Enterprises (BUMN), as regulated by Article 87 of Law Number 30 of 2014 concerning State Administration, taking into account other relevant laws such as Law Number 28 of 1999 concerning Clean State Officials Free from Corruption, Collusion, and Nepotism, and Law Number 5 of 1986 concerning State Administrative Courts, hold the position of Government Official with various derivative consequences, just like other government positions that must adhere to stricter regulations (Ardiansyah and Erliyana, 2022). The provisions of the law governing state administration and state governance are highly contradictory to Article 9G of Law Number 1 of 2025, which states that “members of the Board of Directors, Board of Commissioners, and Supervisory Board of BUMN are not state officials.”

State-owned enterprises (BUMN), as representatives of business entities owned and managed by the state, have rights and obligations determined by law. Decisions and policies aimed at managing businesses based on the BUMN Law are grounded in the interests and welfare of the people through businesses managed by BUMN. The capital for managing BUMN, sourced from state capital participation and other sources, indicates that all organs, including supervisors and commissioners, are state administrators responsible for the actions and policies governing the operation of BUMN (Ansari, 2019).

The change in legal liability status as mentioned in Article 9G of the BUMN Law creates conflicts of norms and legal uncertainty (Asnawi and Zalesny, 2023). The impact of this change in legal liability in Article 9 will lead to a decline in public trust in BUMN, as well as increasing opportunities for corruption and abuse of power. The application of criminal sanctions for violations in the management of state-owned enterprises (BUMN) that cause losses to BUMN has not had a deterrent effect, and the number of violations is increasing, as outlined in Table 1.

**Table 1:** Criminal cases against BUMN commissioners or directors.

Company name	Case
PT Pertamina	Several directors of PT Pertamina, including Riva Siahaan (President Director of PT Pertamina Patra Niaga) and Yoki Firnandi (President Director of PT Pertamina International Shipping), are implicated in a corruption case in the energy sector, with state losses reaching Rp 1 quadrillion (under investigation).
PT Timah Tbk	Harvey Moeis, the husband of actress Sandra Dewi, has also been named a suspect in the corruption case involving PT Timah Tbk.
PT Garuda Indonesia	The former President Director of PT Garuda Indonesia, Emirsyah Satar, has been named a suspect in the alleged corruption case involving the procurement of CRJ 1000 and ATR 72-600 aircraft.
PT Asabri	Corruption cases also occurred at PT Asabri, involving several directors, including Adam Damiri and Sonny Widjaja.
PT Waskita Karya	Desi Arryani, the former President Director of PT Jasa Marga, is also implicated in the corruption case involving a fictitious project at PT Waskita Karya, where she previously served as head of a division.
PT Pelindo II	RJ Lino, the former President Director of PT Pelindo II, is also implicated in a corruption case related to the procurement of goods.

Source: self-managed, year 2025.

Based on Table 1 above, there is a strong correlation with the change in legal liability under Article 9G, which will create opportunities for violations. The concerning impact of this change in legal liability will hinder the growth of BUMN and become a prolonged burden on the state, ultimately leading to mergers, acquisitions, and/or the dissolution of BUMN. Based on Table 1 above, it can also be shown that the involvement of BUMN directors significantly affects the occurrence of losses for BUMN. Therefore, if there is a change in norms at BUMN to not classify the directors’ mistakes in managing BUMN losses, which were not prevented, as criminal acts. Normative provisions should regulate limited prevention so that an act can be said not to be a financial detriment to the state. An example of limited prevention refers to formulating specific preventive measures taken and clearly regulated forms of prevention that can be classified as not constituting a financial detriment to the state (Woolf, 2008).

Based on Table 2 above, it explains that there are several State-Owned Enterprises (BUMN) that have legal issues and cause state losses. If the regulations governing the prevention and enforcement of actions that harm the state’s finances are not strictly, tightly, and limitedly regulated, it will create new problems and cause even greater state losses. The relationship between the case in Table 2 above and Article 9 Letter G of Law Number 1 of 2025 concerning BUMN can be explained that there is a state loss resulting from the actions and negligence carried out by the directors and has fulfilled the elements of state loss. If it is connected with Article 9 Letter G of Law Number 1 of 2025 concerning BUMN, the actions and negligence carried out by the directors cannot necessarily be subject to criminal penalties, this must fulfill the elements that have been formulated in Law Number 1 of 2025 concerning BUMN, one of which is negligence and the absence of supervision carried out.

**Table 2:** Cases of directors at state-owned companies in Indonesia.

PERUM	Case	Year
Perum Perindo	The Corruption Eradication Committee (KPK) arrested a director in connection with bribery related to fish import quotas.	2019
PT Amarta Karya	Former CEO Catur Prabowo and Finance Director Trisna Sutisna were charged with money laundering (TPPU) related to 60 fictitious subcontractor projects with losses of IDR 46 billion.	2018–2022
PT Krakatau Steel	Former CEO Fazwar Bujang was involved in corruption in the Blast Furnace Complex project which caused the state to lose Rp 6.9 trillion.	2011

Source: independently managed in 2026.

### 4.2 Comparison of the legal liability of directors for company losses in Singapore

Meanwhile, in Singapore, Section 157(3)(a) and (b) of the Singapore Companies Act stipulates that: (a) directors are responsible to the company for any deficit caused by their breach, and (b) the offender may be subject to fines or imprisonment in accordance with the provisions, with the fine not exceeding $5,000 or imprisonment for a period not exceeding one year (Alya Nabita Az-zahra and Sri Bakti Yunari, 2024).

**Table:** 

Duties and responsibilities of the director	Fiduciary Duty: Directors have a duty to act honestly, without conflicts of interest, and with the care and skill reasonably expected for the best interests of the Company.
	Financial Statements: The director is required to ensure the company has accurate financial records and present them at the Annual General Meeting of Shareholders (RUPST).
	Obligations when the Company is Insolvent: Directors must consider the interests of creditors when the company is near bankruptcy and may be liable for debts if they knew or should have known the company was trading unlawfully.
Director personally liable	Breach of Fiduciary Duty: If a director abuses trust, returns profits gained from the breach, or causes company losses due to a breach of trust such as theft, they can be held accountable.
	Unlawful Trading: Directors can be held personally liable for the company’s debts if the company trades fraudulently.
	False Statement: Making fraudulent statements in financial reports is an example of a breach of duty that can lead to personal liability.
Legal consequences	Criminal Penalties: Violations such as theft of company property or misuse of assets can result in criminal charges, with fines of up to $5,000 or imprisonment for up to 1 year.
	Claim for Damages: Directors who cause losses to the company may be required to return the profits they earned or compensate for those losses.
	Limitation of Liability: A clause in the articles of association that attempts to indemnify directors for negligence, breach of contract, or breach of trust is invalid and considered null and void.

### 4.3 Comparison of directors’ legal liability for company losses in Malaysia

In Malaysia, company directors are generally not personally liable for the debts and losses of the company because the company is considered a separate legal entity. However, they can be held personally accountable if the losses are caused by errors or omissions in the performance of their duties, or if they provide personal guarantees for the debt. To avoid personal liability, directors must be able to prove that they acted in good faith, with due diligence, care, and have taken steps to prevent the losses.

**Table:** 

General principles	The company has a legal identity separate from its directors and shareholders. Therefore, generally, directors are not liable for the company’s debts or losses.
	Directors can be held personally liable for company losses if there is any error or omission in the performance of their duties, including: 1) Negligence: Not performing duties with good faith, due care, and proper responsibility; 2) Conflict of Interest: The existence of personal interests that conflict with the company’s interests, causing losses; 3) Failure to Take Preventative Action: Failing to take reasonable steps to prevent or address losses; 4) Personal Guarantee: If the director provides a personal guarantee for the company’s debt and the company goes bankrupt.

### 4.4 Comparison of legal responsibility of directors for company losses in Indonesia based on article 9G of law number 1 of 2025

Prior to (the Old State-Owned Enterprises Law) Law Number 19 of 2003 concerning State-Owned Enterprises, directors were often viewed as part of state administrators (quasi-public officials). Losses to state-owned enterprises were often considered state losses and were easily linked to criminal acts of corruption. Following Law No. 1 of 2025, non-state-owned enterprise directors are more firmly positioned as corporate entities. Losses at BUMN do not automatically constitute state losses. This change could create legal loopholes for directors and commissioners to commit violations that result in state financial losses (Jones, 2006). According to Law Number 1 of 2004 concerning State Treasury, Article 1 number 22 states that state losses are a shortage of money, securities and goods of a real and definite amount as a result of unlawful acts, whether intentional or negligent. Perbandingan pada negara dan Malaysia and Singapore were conducted to see the consistency of criminal law enforcement against perpetrators or actions carried out by directors that cause state losses in state-owned enterprises by emphasizing fines without eliminating the criminal element.

## 5 The influence of legal liability for state losses caused by the actions of BUMN directors

The 1945 Constitution of the Republic of Indonesia, Article 4 paragraph (1), grants the President the authority to exercise governmental power to achieve the state’s goal, which is to prosper the people. These provisions are further detailed in Article 33, which serves as the basis for the implementation of constitutional duties for all components of the nation, including state-owned enterprises. Many parties believe that state-owned enterprises are a remarkable economic force and driver. In Singapore and Malaysia, state-owned enterprises (BUMN) make a significant contribution to economic activity. In Indonesia, BUMN operate in a diverse range of sectors or business areas, from banking, energy, food, and infrastructure to transportation, including sea, land, and air (Juliani, 2016).

The Business Judgement Rule doctrine is a doctrine known in corporate law as an immunity for directors in making decisions for the company. This doctrine is the only solution that can be used to protect directors acting in good faith from corporate lawsuits by shareholders or creditors regarding losses arising from decisions made by the directors (Masrurah, 2019).

The Business Judgement Rule principle must meet the classification of its actions so that the Board of Directors can be protected for its decisions (Irianto and Wardani, 2023). The Board of Directors’ decision regarding their negligence, which resulted in losses for the company, means that the directors can be held legally accountable under administrative, civil, and criminal law (Dewi and Harimurti, 2024). Referring to Law Number 1 of 2025 concerning State-Owned Enterprises in Article 9G, the criminal law status, particularly in the Corruption Eradication Law, regarding members of the Board of Directors is not part of the legal subject. This is because a Director’s concern about negligence in their actions that causes harm to the Company could be classified as an act detrimental to state finances and could be subject to the Anti-Corruption Law (Akinsola and Hamzah, 2025).

The absolute application of the Business Judgement Rule principle in the BUMN Law can lead to abuse of authority by members of the board of directors. The concept of the Business Judgement Rule cannot be the basis for releasing the legal status of board members as state officials. The case of Salomon v Salomon & Co Ltd. (1897) provides insight into the disregard for the Business Judgement Rule (Dewi, 2018). The approach used by the court will disregard the usual immunity of corporate officers in this case using the principle of piercing the corporate veil. The concept of limited liability is one of the fundamental instruments in corporate law. This concept is inseparable from the principle of piercing the corporate veil, which can be interpreted as “lifting the veil or curtain of the company.” This concept can be applied not only to shareholders and management or Directors and even Commissioners of the Company, but also to the company itself as an independent legal entity with standing in court (Pickering, 1968). This is based on the awareness that a corporation can be used as a tool for malicious or fraudulent purposes.

There are several data cases that resulted in negligence by directors or commissioners who have been found guilty by the court due to negligence that resulted in their failure to prevent acts or actions that resulted in state losses.

Based on the legal cases that have been decided as in the case below:

a) The President Director of PT Merpati Nusantara Airlines, Hotasi D. P. Nababan, was found guilty by the Supreme Court (Supreme Court Decision No. 2405 K/Pid.Sus/2016). The Supreme Court (MA) holds the view that the wealth of state-owned enterprises (Persero) remains subject to the Anti-Corruption Law because it is part of the separated state finances.
b) Former President Director of PT Pertamina, Karen Galaila Agustiawan, was found guilty of making investments that caused state financial losses amounting to Rp 568 billion.

Based on case 1 above, it can be consistently concluded that both the commissioners and the board of directors have committed unlawful acts due to negligence, resulting in state losses and can be held accountable under both criminal and civil law. The above can serve as an indicator to limit the interpretation that the board of directors or commissioners cannot be considered as causing state losses. Through a real case example, it is explained that the abuse of authority and legal violations that harm state finances cannot be considered merely as ordinary business risks. The Supreme Court affirmed that the wealth of state-owned enterprises (BUMN) remains state assets, so any financial losses arising from negligence or intent can be prosecuted under the Corruption Eradication Law. This explanation emphasises that leaders of public companies have strict legal obligations to maintain the operational integrity and assets of the government. Thus, this judicial decision serves as a legal warning for company officials to manage public funds responsibly.

### 5.1 Classification of state losses in the management of state-owned enterprises

There are differing interpretations regarding legal liability for losses in the management of State-Owned Enterprises (BUMN) that result in financial losses for the state. Based on Law Number 1 of 2025 concerning BUMN, which states that losses in the management of BUMN are not considered financial losses for the state, but on the other hand, a portion of BUMN capital comes from state finances. The inseparable nature of state finances within state-owned enterprises (BUMN) is based on the main elements driving BUMN, which originate from the state: (a) The management of BUMN is appointed by the Minister based on the President’s appointment; (b) The capital for managing BUMN comes from state finances. The protection that must be given to BUMN managers is not to grant legal immunity to BUMN organs, but rather to protect the BUMN themselves in the form of:

a) Training for BUMN managers to avoid losses in BUMN;
b) Improving production quality at the state-owned enterprise;
c) Ensuring acceptable results for state-owned enterprises in all markets, both national and international.

As stated in H. A Deemersemen’s Doctrine of the Autonomy of Material Criminal Law, criminal law has the autonomy to provide definitions different from those found in other branches of law. However, unless otherwise specified by criminal law, the definitions found in other branches of law are used. Regarding state finances and their scope, which have been outlined in the State Finance Law and the State Audit Board Law, and are then defined in the General Explanation of Law Number 31 of 1999 concerning the Eradication of Corruption Crimes (Tipikor), which states: “The state finances referred to are all state wealth in any form, whether separated or not, including all parts of state wealth and all rights and obligations arising from: (a) being under the control, management, and accountability of state institution officials at both the central and regional levels; (b) being under the control, management, and accountability of State-Owned Enterprises/Regional-Owned Enterprises, foundations, legal entities, and companies that include state capital, or companies that include third-party capital based on agreements with the State.” Therefore, it is clear that the phrase “state losses” has a fundamental difference from “state financial losses” in Article 2 paragraph (1) and Article 3 of the Corruption Crimes Eradication Law.

It needs to be emphasised that this article does not mention, regulate, or refer to the applicability of the Anti-Corruption Law at all (Barafi et al., 2022). This means that the existence of this article cannot be interpreted as revoking or suspending the applicability of the Anti-Corruption Law (Teachout, 2008). Moreover, the Anti-Corruption Law explicitly includes an explanation of the broad and substantive scope of “state finances,” which encompasses state assets separated into BUMN. Therefore, the Anti-Corruption Law remains valid and binding, and is not subject to the sectoral administrative regulations stipulated in the BUMN Law. State losses as referred to in Law Number 1 of 2025 concerning State-Owned Enterprises must be understood as part of administrative regulations that emphasise the severance of the legal relationship between state assets and State-Owned Enterprises as private legal entities, through the principle of separated state assets. This concept is used in the context of corporate governance and asset management, and is not directly related to the criminal law regime (corruption; Abdullah and Said, 2018).

Conversely, financial losses to the state in the Anti-Corruption Law are an element of criminal law that refers to any state wealth controlled, owned, or managed by bodies whose funds come from state finances (the state budget/regional budget), including state-owned enterprises through state capital participation. This definition is explicitly affirmed in the general explanation of the Anti-Corruption Law and is substantive, not merely administrative. Therefore, criminal law enforcement is not subject to sectoral administrative limitations, and the Anti-Corruption Law remains independently applicable and binding as a lex specialis in combating corruption in Indonesia.

In Article 4B, which states that the profits or losses experienced by BUMN are the profits or losses of the BUMN. Furthermore, the explanatory section states, “The capital and assets of BUMN belong to the BUMN, and any profits or losses experienced by BUMN are not the profits or losses of the state.” The profits or losses of BUMN include, but are not limited to, the profits or losses of BUMN arising from the management of some or all of the BUMN assets in investment and/or operational activities of the relevant BUMN. The Law Number 1 of 2025 concerning State-Owned Enterprises itself does not provide a definition of state losses. To apply and classify criminal acts within the State-Owned Enterprises Law, the elements of accountability for actions in the management of State-Owned Enterprises or policies taken can be determined, including:

1) There was negligence caused by not following standard processes in managing state-owned enterprises;
2) There are transactional aspects in the management of BUMN, which indicates that they benefit certain groups (Vagliasindi, 2008);
3) The operation of state-owned enterprises not based on the public interest.
4) State-owned enterprises (BUMN) incur losses due to policies not based on the principles and objectives of BUMN management (Ansari, 2019);
5) The management of state-owned enterprises (BUMN) creates conflicts with laws and regulations that are interconnected;
6) There are indications of policies that cause social conflict in the management of BUMN, particularly regarding land ownership and the transfer of land rights;

Based on the six classifications of actions above, all BUMN organs can be held criminally liable for corruption, money laundering, and gratuities. Changes to the State-Owned Enterprises Law must receive immediate attention from the government to avoid losses for BUMN or ineffective management of BUMN through the formulation of specific provisions designed to provide legal certainty in seeking criminal legal accountability against BUMN organs.

Legal liability, whether criminal, civil, or administrative, can be applied as long as the Board of Directors is proven to have committed the actions. Harmonisation of laws related to the management of BUMN must be carried out, particularly concerning the status of BUMN as legal subjects equal to other legal subjects, namely individuals and private corporations, to ensure legal certainty in the application of the law regarding the legal accountability of the Board of Directors.

## 6 Conclusion

The reformulation of Article 9, Letter G of Law Number 1 of 2025 concerning State-Owned Enterprises (BUMN) can prevent abuse of authority due to sanctions for violations that are not deterrent for BUMN directors. Based on research findings, Article 9G of the BUMN Law has limited the meaning of criminal acts to actions of directors that cause losses to BUMN finances. Based on research findings, there are no clear indicators to determine criminal acts that can be applied to BUMN directors that cause state losses. From these research findings, it is important to formulate strict and limited indicators of actions or deeds of directors that can be clearly categorized as criminal acts or outside the realm of criminal law. The limited meaning formulated in its group aims to prevent broad interpretations and prevent inconsistencies in the application of the article. Inconsistency in law enforcement in preventing and handling actions that can cause state financial losses will cause discrimination against similar actions. Changes to sanctions against elements of violations committed by directors or commissioners must be formulated clearly to avoid double interpretations in their application by formulating a limited definition of punishable violations.

## Data Availability

The datasets presented in this study can be found in online repositories. The names of the repository/repositories and accession number(s) can be found in the article/supplementary material.
